# Crystal structure of bis­(aceto­nitrile-κ*N*)bis­(4-benzoyl­pyridine-κ*N*)bis­(thio­cyanato-κ*N*)cobalt(II)

**DOI:** 10.1107/S2056989017002201

**Published:** 2017-02-14

**Authors:** Stefan Suckert, Julia Werner, Inke Jess, Christian Näther

**Affiliations:** a Institut für Anorganische Chemie, Christian-Albrechts-Universität Kiel, Max-Eyth Strasse 2, D-24118 Kiel, Germany

**Keywords:** crystal structure, discrete complex, cobalt(II) thio­cyanate, 4-benzoyl­pyridine, hydrogen bonding

## Abstract

The crystal structure of the title compound, [Co(NCS)_2_(C_2_H_3_N)_2_(C_12_H_9_NO)_2_], consists of cobalt(II) cations that are octa­hedrally coordinated by the N atoms of two terminal thio­cyanate anions, two aceto­nitrile mol­ecules and two 4-benzoyl­pyridine ligands. The discrete complexes are located on centres of inversion. They are connected by weak inter­molecular C—H⋯O and C—H⋯S hydrogen-bonding inter­actions between one of the pyridine H atoms and the carbonyl O atom, and between one of the methyl H atoms of the aceto­nitrile mol­ecule and the thio­cyanate S atoms into layers parallel to (101). No pronounced inter­molecular inter­actions are observed between these layers.

## Abstract 2

The crystal structure of the title compound consists of discrete octa­hedral complexes, that are linked by inter­molecular C—H⋯O and C—H⋯S hydrogen bonding into layers.

## Chemical context   

In recent times, the synthesis of materials exhibiting cooperative magnetic properties has still been a topic of major inter­est in coordination chemistry (Zhang *et al.*, 2011 ▸). A good approach for the preparation of such compounds is the use of small anionic ligands such as *e.g.* thio­cyanate anions to link paramagnetic cations, enabling a magnetic exchange between the cations (Palion-Gazda *et al.*, 2015 ▸; Massoud *et al.*, 2013 ▸). During the last few years, our group has reported on a number of coordination polymers with thio­cyanato ligands that show different magnetic phenomena, including a slow relaxation of the magnetization (Werner *et al.*, 2014 ▸, 2015*a* ▸,*b* ▸,*c* ▸,*d* ▸). In the course of this project, we became inter­ested in compounds based on 4-benzoyl­pyridine, for which at that time only three thio­cyanato compounds had been reported (Drew *et al.*, 1985 ▸; Soliman *et al.*, 2014 ▸; Bai *et al.*, 2011 ▸). During these investigations, we obtained a compound with composition [Co(NCS)_2_(4-benzoyl­pyridine)_2_] in which the Co^II^ cations are linked by pairs of anionic ligands into chains. In contrast to all other such chain compounds where all ligands are always *trans*-coordinating, in this compound a *cis*-coordination of the N and the S atoms of the thio­cyanate anions was observed (Rams *et al.*, 2017 ▸). Therefore, we assumed that this compound might be metastable and that a second modification with the usual *trans-*coordination could be prepared by thermal annealing of precursors with terminal N-bonded thio­cyanate anions. In this context, it is noted that there are many examples where different modifications or isomers have been obtained by this alternative route (Werner *et al.*, 2015*a* ▸,*c* ▸; Suckert *et al.*, 2016 ▸). In the course of these studies, crystals of the title compound, [Co(NCS)_2_(C_2_H_3_N)_2_(C_12_H_9_NO)_2_], were obtained and characterized by single crystal X-ray diffraction. Unfortunately, no pure crystalline powder could be obtained, which prevented further investigations of the thermal properties of this compound.
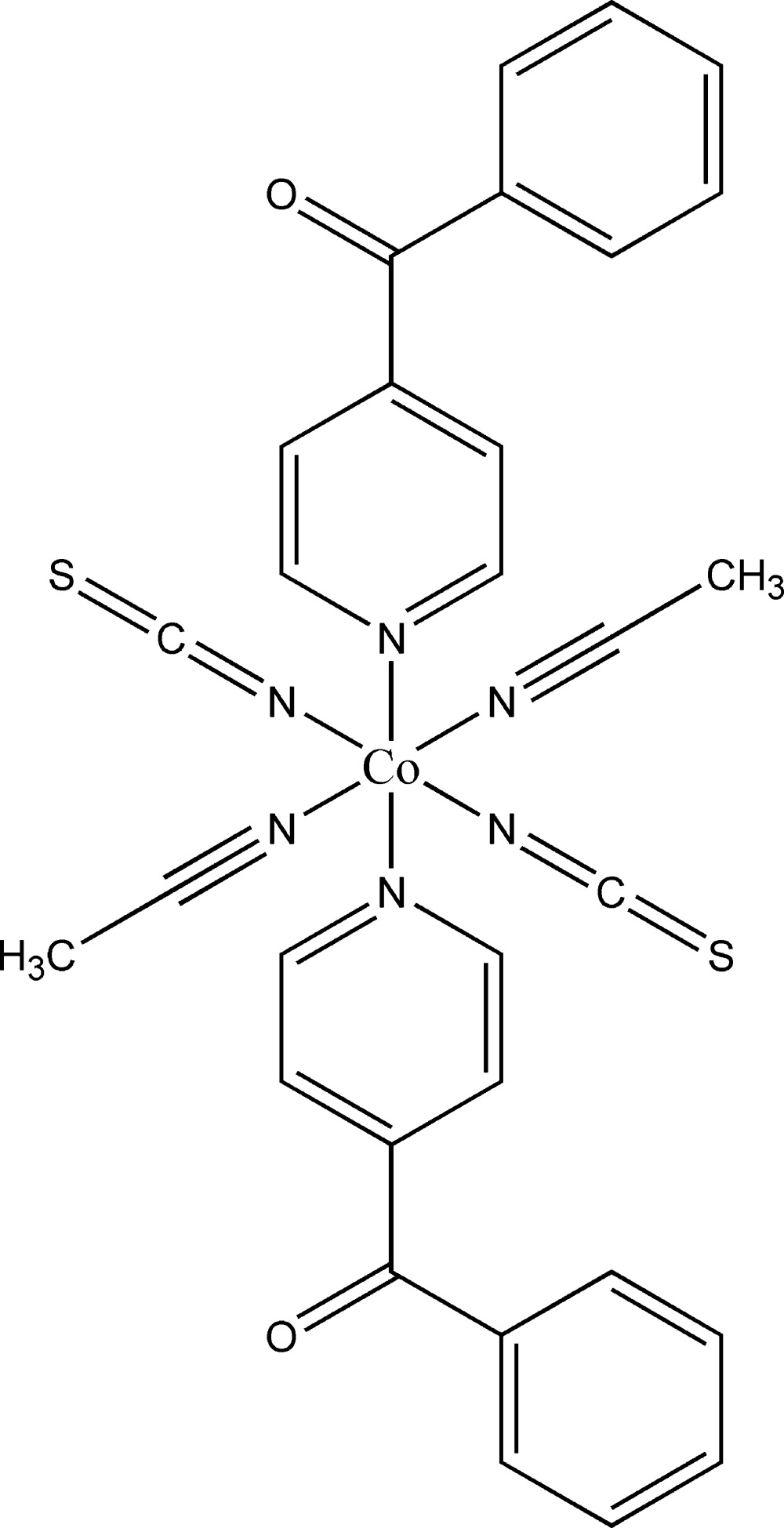


## Structural commentary   

**Figure 1:**
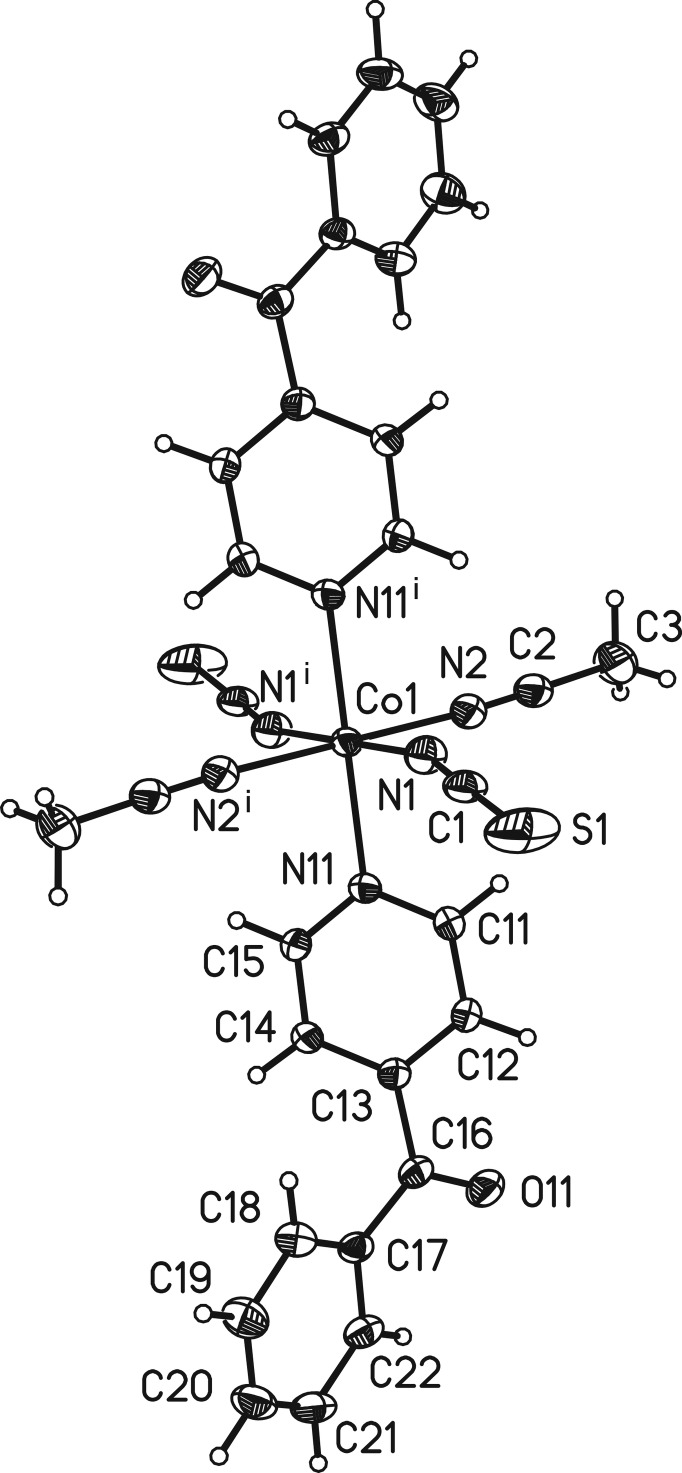
View of a discrete complex of the title compound, showing the atom-labelling scheme. Displacement ellipsoids are drawn at the 50% probability level. [Symmetry code: (i) −*x* + 1, −*y*, −*z* + 1.]

The asymmetric unit of the title compound consists of one cobalt(II) cation, one thio­cyanato anion, one aceto­nitrile mol­ecule and one neutral 4-benzoyl­pyridine ligand. The cobalt(II) cation is located on a center of inversion while the thio­cyanato anion, the aceto­nitrile mol­ecule and the 4-benzoyl­pyridine ligand are located in general positions. The Co^II^ cation is octa­hedrally coordinated by the N atoms of two terminal anionic ligands, two aceto­nitrile mol­ecules and two 4-benzoyl­pyridine ligands (Fig. 1 ▸). As expected, the Co—N bond lengths to the thio­cyanate anions are significantly shorter [2.0520 (15) Å] than those to the pyridine N atom of the neutral 4-benzoyl­pyridine ligand [2.1831 (13) Å]. All bond lengths are in agreement with values reported in the literature (Drew *et al.*, 1985 ▸; Soliman *et al.*, 2014 ▸). The 4-benzoyl­pyridine ligand is not planar; the dihedral angle between the phenyl and pyridine rings is 55.37 (8)°. This is in agreement with values retrieved from the literature, which vary between 40.4 and 74.3° (Escuer *et al.*, 2000 ▸, 2004 ▸).

## Supra­molecular features   

**Figure 2:**
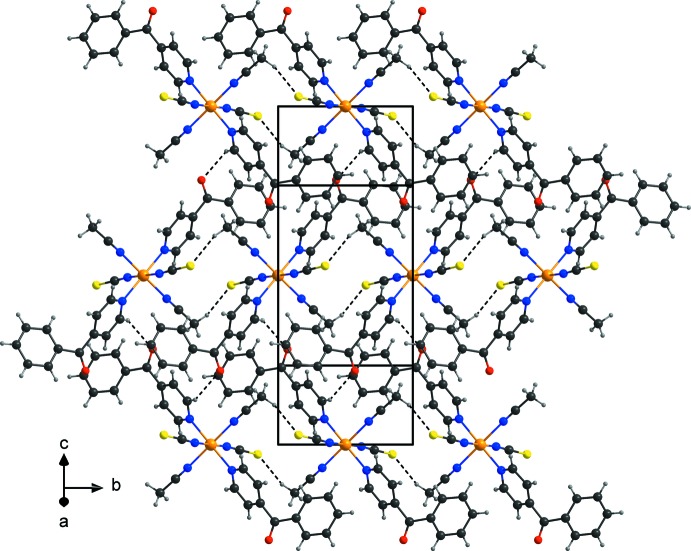
View of the hydrogen-bonded layers extending parallel to (101). Hydrogen bonds are shown as dashed lines.

**Figure 3:**
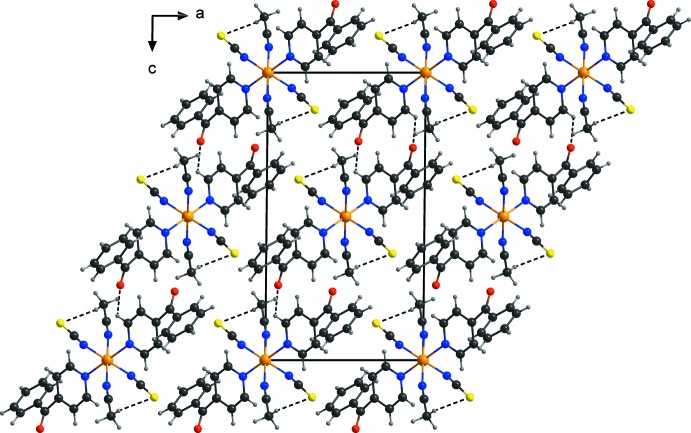
Part of the crystal structure of the title compound, showing the hydrogen-bonded layers. Hydrogen bonds are shown as dashed lines.

In the crystal structure of the title compound, the discrete complexes are linked by inter­molecular C—H⋯O hydrogen bonds between one of the pyridine ring H atoms and the oxygen atom of the 4-benzoyl­pyridine ligand of a neighboring complex into dimers, which are further connected into chains (Fig. 2 ▸, Table 1 ▸). These chains are further linked into layers parallel to (101) by centrosymmetric pairs of inter­molecular C—H⋯S hydrogen bonds between one of the aceto­nitrile hydrogen atoms and the neighbouring thio­cyanato S atom (Fig. 3 ▸, Table 1 ▸). Pronounced inter­molecular inter­actions are not observed between these layers.

## Database survey   

To the best of our knowledge, there are only three coordination compounds with thio­cyanato ligands and with 4-benzoyl­pyridine reported in the Cambridge Structural Database (Version 5.38, last update 2016; Groom *et al.*, 2016 ▸). In two of these structures, Co^II^ or Ni^II^ cations are octa­hedrally coordinated by four 4-benzoyl­pyridine ligands and two thio­cyanate anions (Drew *et al.*, 1985 ▸; Soliman *et al.*, 2014 ▸). In the third compound, Cu^II^ cations are coordinated in a square-planar mode by two 4-benzoyl­pyridine ligands and two thio­cyanate anions (Bai *et al.*, 2011 ▸). A general search for coord­ination compounds with 4-benzoyl­pyridine resulted in 22 structures including the aforementioned ones. One of these compounds consists of Mn^II^ cations that are octa­hedrally coordinated by two 4-benzoyl­pyridine ligands as well as by four *μ*_1,3_-bridging azido ligands and linked into chains by the anionic ligands (Mautner *et al.*, 2015 ▸).

## Synthesis and crystallization   

Co(NCS)_2_ and 4-benzoyl­pyridine were purchased from Alfa Aesar. Crystals of the title compound suitable for single crystal X-ray diffraction were obtained by the reaction of 26.3 mg Co(NCS)_2_ (0.15 mmol) with 55.0 mg 4-benzoyl­pyridine (0.3 mmol) in aceto­nitrile (1.5 ml) after a few days.

## Refinement   

Crystal data, data collection and structure refinement details are summarized in Table 2 ▸. The C-bound H atoms were positioned with idealized geometry and were refined with fixed isotropic displacement parameters *U*_iso_(H) = 1.2*U*_eq_(C) for aromatic and *U*_iso_(H) = 1.5 *U*_eq_(C) for methyl H atoms using a riding model. The methyl H atoms were allowed to rotate but not to tip.

## Supplementary Material

Crystal structure: contains datablock(s) I. DOI: 10.1107/S2056989017002201/wm5365sup1.cif

Structure factors: contains datablock(s) I. DOI: 10.1107/S2056989017002201/wm5365Isup2.hkl

CCDC reference: 1532114

Additional supporting information:  crystallographic information; 3D view; checkCIF report

## Figures and Tables

**Table 1:** Hydrogen-bond geometry (Å, °)

*D*—H⋯*A*	*D*—H	H⋯*A*	*D*⋯*A*	*D*—H⋯*A*
C3—H3*A*⋯S1^i^	0.98	2.85	3.771 (3)	156
C11—H11⋯O11^ii^	0.95	2.49	3.193 (2)	131

Symmetry codes: (i) 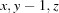; (ii) 
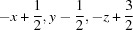
.

**Table 2:** Experimental details

Crystal data	
Chemical formula	[Co(NCS)_2_(C_2_H_3_N)_2_(C_12_H_9_NO)_2_]
*M*_r_	623.60
Crystal system, space group	Monoclinic, *P*2_1_/*n*
Temperature (K)	200
*a*, *b*, *c* (Å)	10.0304 (6), 8.3355 (4), 18.2581 (12)
β (°)	90.547 (8)
*V* (Å^3^)	1526.46 (15)
*Z*	2
Radiation type	Mo *K*α
μ (mm^−1^)	0.74
Crystal size (mm)	0.16 × 0.08 × 0.02

Data collection	
Diffractometer	Stoe IPDS1
Absorption correction	Numerical (*X-SHAPE* and *X-RED32*; Stoe, 2008 ▸)
*T*_min_, *T*_max_	0.897, 0.964
No. of measured, independent and observed [*I* > 2σ(*I*)] reflections	17991, 3347, 2895
*R*_int_	0.032
(sin θ/λ)_max_ (Å^−1^)	0.640

Refinement	
*R*[*F* ^2^ > 2σ(*F* ^2^)], *wR*(*F* ^2^), *S*	0.036, 0.096, 1.04
No. of reflections	3347
No. of parameters	189
H-atom treatment	H-atom parameters constrained
Δρ_max_, Δρ_min_ (e Å^−3^)	0.46, −0.69

Computer programs: *X-AREA* (Stoe, 2008 ▸), *SHELXS97* (Sheldrick, 2008 ▸), *SHELXL2014* (Sheldrick, 2015 ▸), *XP* in *SHELXTL* (Sheldrick, 2008 ▸), *DIAMOND* (Brandenburg, 1999 ▸) and *publCIF* (Westrip, 2010 ▸).

## supplementary crystallographic information

### Crystal data

**Table:**

[Co(NCS)_2_(C_2_H_3_N)_2_(C_12_H_9_NO)_2_]	*F*(000) = 642
*M**_r_* = 623.60	*D*_x_ = 1.357 Mg m^−^^3^
Monoclinic, *P*2_1_/*n*	Mo *K*α radiation, λ = 0.71073 Å
*a* = 10.0304 (6) Å	Cell parameters from 17991 reflections
*b* = 8.3355 (4) Å	θ = 2.7–27.1°
*c* = 18.2581 (12) Å	µ = 0.74 mm^−^^1^
β = 90.547 (8)°	*T* = 200 K
*V* = 1526.46 (15) Å^3^	Block, purple
*Z* = 2	0.16 × 0.08 × 0.02 mm

### Data collection

**Table:**

Stoe IPDS-1 diffractometer	2895 reflections with *I* > 2σ(*I*)
phi scans	*R*_int_ = 0.032
Absorption correction: numerical (*X-SHAPE* and *X-RED32*; Stoe, 2008)	θ_max_ = 27.1°, θ_min_ = 2.7°
*T*_min_ = 0.897, *T*_max_ = 0.964	*h* = −12→12
17991 measured reflections	*k* = −10→10
3347 independent reflections	*l* = −23→23

### Refinement

**Table:**

Refinement on *F*^2^	Hydrogen site location: inferred from neighbouring sites
Least-squares matrix: full	H-atom parameters constrained
*R*[*F*^2^ > 2σ(*F*^2^)] = 0.036	*w* = 1/[σ^2^(*F*_o_^2^) + (0.0604*P*)^2^ + 0.5217*P*] where *P* = (*F*_o_^2^ + 2*F*_c_^2^)/3
*wR*(*F*^2^) = 0.096	(Δ/σ)_max_ < 0.001
*S* = 1.04	Δρ_max_ = 0.46 e Å^−^^3^
3347 reflections	Δρ_min_ = −0.69 e Å^−^^3^
189 parameters	Extinction correction: SHELXL2014 (Sheldrick, 2015), Fc^*^=kFc[1+0.001xFc^2^λ^3^/sin(2θ)]^-1/4^
0 restraints	Extinction coefficient: 0.030 (3)

### Special details

**Table:**

Geometry. All esds (except the esd in the dihedral angle between two l.s. planes) are estimated using the full covariance matrix. The cell esds are taken into account individually in the estimation of esds in distances, angles and torsion angles; correlations between esds in cell parameters are only used when they are defined by crystal symmetry. An approximate (isotropic) treatment of cell esds is used for estimating esds involving l.s. planes.

### Fractional atomic coordinates and isotropic or equivalent isotropic displacement parameters (Å^2^)

**Table:**

	*x*	*y*	*z*	*U*_iso_*/*U*_eq_	
Co1	0.5000	0.0000	0.5000	0.02109 (12)	
N1	0.65155 (15)	0.11822 (18)	0.55359 (9)	0.0322 (3)	
C1	0.71187 (17)	0.2142 (2)	0.58531 (10)	0.0313 (4)	
S1	0.79948 (6)	0.34828 (9)	0.62877 (5)	0.0698 (3)	
N2	0.49639 (16)	−0.17253 (18)	0.58858 (8)	0.0311 (3)	
C2	0.50846 (19)	−0.2619 (2)	0.63506 (10)	0.0317 (4)	
C3	0.5267 (3)	−0.3769 (3)	0.69412 (13)	0.0514 (6)	
H3A	0.5739	−0.4714	0.6757	0.077*	
H3B	0.4395	−0.4093	0.7128	0.077*	
H3C	0.5791	−0.3276	0.7337	0.077*	
N11	0.35979 (14)	0.15671 (16)	0.55624 (7)	0.0234 (3)	
C11	0.37171 (18)	0.1751 (2)	0.62899 (9)	0.0281 (4)	
H11	0.4386	0.1159	0.6543	0.034*	
C12	0.29053 (18)	0.2770 (2)	0.66876 (9)	0.0283 (4)	
H12	0.3009	0.2853	0.7204	0.034*	
C13	0.19360 (17)	0.36697 (18)	0.63229 (9)	0.0240 (3)	
C14	0.18049 (17)	0.3477 (2)	0.55676 (9)	0.0266 (3)	
H14	0.1153	0.4065	0.5299	0.032*	
C15	0.26444 (17)	0.2409 (2)	0.52146 (9)	0.0262 (3)	
H15	0.2538	0.2267	0.4701	0.031*	
C16	0.10657 (17)	0.4749 (2)	0.67758 (9)	0.0253 (3)	
C17	0.06203 (16)	0.63359 (18)	0.64974 (9)	0.0238 (3)	
C18	0.13193 (17)	0.7173 (2)	0.59627 (10)	0.0296 (4)	
H18	0.2059	0.6687	0.5729	0.036*	
C19	0.0930 (2)	0.8723 (2)	0.57732 (12)	0.0390 (4)	
H19	0.1417	0.9300	0.5416	0.047*	
C20	−0.0159 (2)	0.9428 (2)	0.61001 (12)	0.0396 (5)	
H20	−0.0419	1.0484	0.5966	0.048*	
C21	−0.08743 (19)	0.8590 (2)	0.66259 (11)	0.0361 (4)	
H21	−0.1631	0.9070	0.6845	0.043*	
C22	−0.04865 (17)	0.7059 (2)	0.68300 (10)	0.0293 (4)	
H22	−0.0968	0.6497	0.7195	0.035*	
O11	0.07747 (15)	0.43077 (16)	0.73898 (7)	0.0373 (3)	

### Atomic displacement parameters (Å^2^)

**Table:**

	*U*^11^	*U*^22^	*U*^33^	*U*^12^	*U*^13^	*U*^23^
Co1	0.02286 (18)	0.01851 (17)	0.02185 (17)	0.00227 (11)	−0.00268 (11)	−0.00305 (10)
N1	0.0281 (7)	0.0316 (8)	0.0370 (8)	−0.0008 (6)	−0.0050 (6)	−0.0088 (6)
C1	0.0233 (8)	0.0312 (9)	0.0393 (9)	0.0064 (7)	−0.0022 (7)	−0.0102 (7)
S1	0.0407 (3)	0.0604 (4)	0.1081 (6)	−0.0008 (3)	−0.0150 (3)	−0.0554 (4)
N2	0.0377 (8)	0.0274 (7)	0.0281 (7)	0.0052 (6)	0.0022 (6)	0.0011 (6)
C2	0.0358 (9)	0.0264 (8)	0.0330 (9)	0.0051 (7)	0.0027 (7)	−0.0012 (7)
C3	0.0766 (17)	0.0344 (11)	0.0432 (11)	0.0146 (10)	0.0073 (11)	0.0130 (9)
N11	0.0255 (7)	0.0208 (6)	0.0239 (6)	0.0034 (5)	−0.0014 (5)	−0.0021 (5)
C11	0.0338 (9)	0.0275 (8)	0.0230 (8)	0.0076 (7)	−0.0034 (7)	0.0008 (6)
C12	0.0371 (9)	0.0287 (8)	0.0192 (7)	0.0064 (7)	−0.0013 (6)	−0.0004 (6)
C13	0.0280 (8)	0.0210 (7)	0.0232 (7)	0.0014 (6)	0.0001 (6)	−0.0010 (6)
C14	0.0279 (8)	0.0277 (8)	0.0240 (8)	0.0078 (6)	−0.0048 (6)	−0.0031 (6)
C15	0.0292 (8)	0.0281 (8)	0.0211 (7)	0.0053 (6)	−0.0048 (6)	−0.0044 (6)
C16	0.0283 (8)	0.0241 (7)	0.0235 (7)	−0.0008 (6)	0.0000 (6)	−0.0058 (6)
C17	0.0229 (8)	0.0223 (7)	0.0262 (8)	0.0002 (6)	−0.0023 (6)	−0.0068 (6)
C18	0.0278 (8)	0.0250 (8)	0.0361 (9)	0.0009 (6)	0.0023 (7)	−0.0016 (7)
C19	0.0414 (11)	0.0280 (9)	0.0477 (11)	0.0002 (8)	0.0017 (9)	0.0040 (8)
C20	0.0456 (11)	0.0236 (8)	0.0495 (11)	0.0083 (8)	−0.0084 (9)	−0.0052 (8)
C21	0.0304 (9)	0.0328 (9)	0.0450 (11)	0.0082 (7)	−0.0051 (8)	−0.0162 (8)
C22	0.0265 (8)	0.0308 (8)	0.0305 (8)	0.0003 (7)	0.0008 (7)	−0.0108 (7)
O11	0.0517 (8)	0.0352 (7)	0.0254 (6)	0.0055 (6)	0.0083 (6)	−0.0005 (5)

### Geometric parameters (Å, º)

**Table:**

Co1—N1^i^	2.0520 (15)	C13—C14	1.393 (2)
Co1—N1	2.0520 (15)	C13—C16	1.506 (2)
Co1—N2	2.1647 (15)	C14—C15	1.388 (2)
Co1—N2^i^	2.1648 (15)	C14—H14	0.9500
Co1—N11^i^	2.1831 (13)	C15—H15	0.9500
Co1—N11	2.1831 (13)	C16—O11	1.218 (2)
N1—C1	1.155 (2)	C16—C17	1.485 (2)
C1—S1	1.6244 (18)	C17—C18	1.394 (2)
N2—C2	1.135 (2)	C17—C22	1.406 (2)
C2—C3	1.453 (3)	C18—C19	1.392 (3)
C3—H3A	0.9800	C18—H18	0.9500
C3—H3B	0.9800	C19—C20	1.380 (3)
C3—H3C	0.9800	C19—H19	0.9500
N11—C15	1.341 (2)	C20—C21	1.392 (3)
N11—C11	1.341 (2)	C20—H20	0.9500
C11—C12	1.386 (2)	C21—C22	1.384 (3)
C11—H11	0.9500	C21—H21	0.9500
C12—C13	1.392 (2)	C22—H22	0.9500
C12—H12	0.9500		
			
N1^i^—Co1—N1	180.0	C11—C12—H12	120.3
N1^i^—Co1—N2	91.13 (6)	C13—C12—H12	120.3
N1—Co1—N2	88.88 (6)	C12—C13—C14	118.03 (15)
N1^i^—Co1—N2^i^	88.87 (6)	C12—C13—C16	117.70 (14)
N1—Co1—N2^i^	91.13 (6)	C14—C13—C16	124.24 (15)
N2—Co1—N2^i^	180.0	C15—C14—C13	118.79 (15)
N1^i^—Co1—N11^i^	88.05 (6)	C15—C14—H14	120.6
N1—Co1—N11^i^	91.95 (6)	C13—C14—H14	120.6
N2—Co1—N11^i^	88.24 (5)	N11—C15—C14	123.27 (15)
N2^i^—Co1—N11^i^	91.76 (5)	N11—C15—H15	118.4
N1^i^—Co1—N11	91.95 (6)	C14—C15—H15	118.4
N1—Co1—N11	88.05 (6)	O11—C16—C17	120.68 (15)
N2—Co1—N11	91.76 (5)	O11—C16—C13	118.05 (15)
N2^i^—Co1—N11	88.24 (5)	C17—C16—C13	121.22 (14)
N11^i^—Co1—N11	180.00 (5)	C18—C17—C22	119.48 (16)
C1—N1—Co1	162.48 (14)	C18—C17—C16	122.27 (15)
N1—C1—S1	178.75 (18)	C22—C17—C16	118.06 (15)
C2—N2—Co1	172.91 (16)	C19—C18—C17	119.76 (17)
N2—C2—C3	178.8 (2)	C19—C18—H18	120.1
C2—C3—H3A	109.5	C17—C18—H18	120.1
C2—C3—H3B	109.5	C20—C19—C18	120.58 (19)
H3A—C3—H3B	109.5	C20—C19—H19	119.7
C2—C3—H3C	109.5	C18—C19—H19	119.7
H3A—C3—H3C	109.5	C19—C20—C21	119.97 (18)
H3B—C3—H3C	109.5	C19—C20—H20	120.0
C15—N11—C11	117.74 (14)	C21—C20—H20	120.0
C15—N11—Co1	123.36 (11)	C22—C21—C20	120.19 (17)
C11—N11—Co1	118.85 (11)	C22—C21—H21	119.9
N11—C11—C12	122.79 (15)	C20—C21—H21	119.9
N11—C11—H11	118.6	C21—C22—C17	120.00 (17)
C12—C11—H11	118.6	C21—C22—H22	120.0
C11—C12—C13	119.35 (15)	C17—C22—H22	120.0

Symmetry code: (i) −*x*+1, −*y*, −*z*+1.

### Hydrogen-bond geometry (Å, º)

**Table:**

*D*—H···*A*	*D*—H	H···*A*	*D*···*A*	*D*—H···*A*
C3—H3*A*···S1^ii^	0.98	2.85	3.771 (3)	156
C11—H11···O11^iii^	0.95	2.49	3.193 (2)	131

Symmetry codes: (ii) *x*, *y*−1, *z*; (iii) −*x*+1/2, *y*−1/2, −*z*+3/2.
